# Interaction of influenza virus NS1 protein with growth arrest-specific protein 8

**DOI:** 10.1186/1743-422X-6-218

**Published:** 2009-12-09

**Authors:** Lixia Zhao, Long Xu, Xiaowei Zhou, Qingyu Zhu, Zhixin Yang, Chuanfu Zhang, Xudong Zhu, Mengbin Yu, Yingying Zhang, Xinghui Zhao, Peitang Huang

**Affiliations:** 1 Laboratory of protein engineering, Beijing Institute of Biotechnology, Beijing 100071, PR China; 2 State key laboratory of pathogen and biosecurity, Beijing Institute of Microbiology and Epidemiology, Beijing 100071, PR China

## Abstract

NS1 protein is the only non-structural protein encoded by the influenza A virus, and it contributes significantly to disease pathogenesis by modulating many virus and host cell processes. A two-hybrid screen for proteins that interact with NS1 from influenza A yielded growth arrest-specific protein 8. Gas8 associated with NS1 *in vitro *and *in vivo*. Deletion analysis revealed that the N-terminal 260 amino acids of Gas8 were able to interact with NS1, and neither the RNA-binding domain nor the effector domain of NS1 was sufficient for the NS1 interaction. We also found that actin, myosin, and drebrin interact with Gas8. NS1 and β-actin proteins could be co-immunoprecipitated from extracts of transfected cells. Furthermore, actin and Gas8 co-localized at the plasma membrane. These results are discussed in relation to the possible functions of Gas8 protein and their relevance in influenza virus release.

## Introduction

Influenza A viral NS1 protein is a multifunctional protein that is capable of both protein-protein and protein-RNA interactions [1]. It binds non-specifically to double-stranded RNA and specifically to protein targets. NS1 binds directly to p85β to activate phosphatidylinositol 3-kinase signaling [2]. It binds a cleavage and polyadenylation specificity factor to inhibit the maturation and export of host antiviral mRNAs, and it inhibits poly(A)-binding protein II [3]. NS1 protein interacts with the viral RNA polymerase complex [4], the eukaryotic translation initiation factor eIF4GI [5], NS1-I [6], NS1-BP [7], Staufen [8], and nucleolin [9]. Association of NS1 protein with host factors may affect apoptosis [10,11].

Growth arrest-specific genes are expressed preferentially in cultured cells that have entered a quiescent state following serum deprivation or growth to confluence. To date, eleven *GAS *genes have been identified that act in a variety of biological functions, including the control of microfilament organization [12], nerve cell growth or differentiation [13], apoptosis [14], tyrosine kinase receptor activity [15], and negative and positive control of the cell cycle [16,17]. No sequence similarity or common structural features have been found among the *GAS *genes or proteins [18].

*GAS8*, also known as *GAS11*, is located at 16q24.3 and was found to be a common deletion present in breast and prostate carcinomas. It was viewed as a potential tumor suppressor gene. The *GAS8 *gene consists of 11 exons spanning approximately 25 kb. Northern blot analysis identified two ubiquitously expressed mRNAs of 3.4 and 1.8 kb in length. Another gene, C16orf3, lies within intron 2 of *GAS8*, and is transcribed in the opposite orientation of *GAS8 *[19].

Gas8 protein associates with microtubules *in vitro *and *in vivo*. Deletion analysis identified a microtubule-binding domain (GMAD) and a region that attenuates Gas8-microtubule interactions (IMAD) [20]. Gas8 homologs in *Trypanosoma brucei *and *Chlamydomonas reinhardtii *are integral components of the flagellar axoneme that regulates flagellar beating. The *GAS8 *gene is also expressed in a variety of mammalian cells that lack motile cilia. In COS7 cells, Gas8 is localized to the Golgi apparatus. This localization is dependent on intact microtubules and is regulated by the cell cycle, as Gas8 is dispersed throughout the cytoplasm as cells progress through mitosis [21].

In adult mice, *GAS8 *mRNA and protein are found predominantly in the testes, where expression is regulated during the post-meiotic development of male gametocytes [22].

We isolated Gas8 in a two-hybrid screen for proteins that interact with the influenza A virus protein NS1. We found that NS1 and Gas8 co-localize. Gas8 also interacted with actin, myosin, and drebrin.

## Materials and methods

### Cells and cell culture

293FT, Hela, CV-1, NIH3T3, and BHK21 cells were maintained in Dulbecco's Modified Eagle's Medium (DMEM) containing 10% heat-inactivated fetal calf serum (HyClone). A549 cells were maintained in McCoy's 5A medium supplemented with 10% heat-inactivated fetal calf serum.

### Construction of plasmids

To generate the NS1 expression construct for the CytoTrap two-hybrid system, cDNAs encoding the NS1 proteins of A/Swine/Colorado/1/77 (H3N2) were amplified using the primers listed in Table 1 and cloned into pSos, creating pSos-NS1. To generate an N-terminally myc-tagged NS1 expression construct, the open reading frames encoding NS1 were amplified using the primers listed in Table 1. The PCR products were cloned into pCMV-Myc via the SalI and NotI restriction sites to create pCMV-Myc-NS1. The expression plasmids pGEX-6P-1-NS1 and pEGFP-N3-NS1 were generated by inserting an NS1 cDNA corresponding to amino acids 1 to 237 between the EcoRI/NotI sites of pGEX-6P-1 and the EcoRI/KpnI sites of pEGFP-N3 (NEB), respectively. The GAS8 gene purchased from Proteintech was cloned by PCR using a primer pair specific for the human cDNA (GeneBank accession no. NM_001481). The products were cloned into pDsRed-Express-C1 and pCMV-Tag 2B respectively, creating pDsRed-GAS8 and FLAG-tagged GAS8. Different NS1 and Gas8 deletion mutants were generated by PCR, as above. All primers are listed in Table 1 and all constructs were confirmed by sequence analysis.

**Table 1:** Primers used in this study

Purpose	Primer sequence (5'-3')	
	Forward	Reverse
NS1 pSos	CGGTCGACGATGGATTCCAACACTGTGT	ACGTGCGGCCGC ATCAGCCATCTTATCTCTTC

NS1 pCMV-Myc	ACGCGTCGACCATGGATTCCAACACTGTGTC	TTGCGGCCGC TCAATCAGCCATCTTATCTC
NS1 pEGFP-N3	CCGAATTCTATGGATTCCAACACTGTGTC	GGGGTACC ATCAGCCATCTTATCTCTTC
NS1 pGEX-6P-1	CCGAATTCATGGATTCCAACACTGTGTC	TTGCGGCCGC TCAATCAGCCATCTTATCTC
NS1_1-80_ pCMV-Myc	ACGCGTCGACC ATGGATTCCAACACTGTGTC	TTGCGGCCGC TCAGGTCATTGTAAGCGCCTC
NS1_81-238_ pCMV-Myc	ACGCGTCGACC ATGGCCTCCACACCTGC	TTGCGGCCGC TCAATCAGCCATCTTATCTC
GAS8 pDsRed-Express-C1	GGAATTCGATGGCACCGAAAAAGAAAGGGAAGA	GGGGTACCCGTCGGGGTGCCCACCAGTCCCGC
GAS8 pCMV-Tag 2B	CGGAATTCATGGCACCGAAAAAGAAAGGGAAGA	CCCTCGAGTTACGTCGGGGTGCCCACCAGTCC
GAS8_1-260_ pCMV-Tag 2B	CGGAATTCATGGCACCGAAAAAGAAAGGGAAGA	CCCTCGAGTTACTTCCGCATGTCCTCCATCTG
GAS8_260-478_ pCMV-Tag 2B	CGGAATTCAAGGAGGACCACCTGGAGAGGG	CCCTCGAGTTACGTCGGGGTGCCCACCAGTCCCGC

### Isolation of NS1-interacting cDNA clones using the yeast interaction trap

The CytoTrap two-hybrid system (Stratagene) was used to identify and isolate CytoTrap XR premade library (Merck) cDNAs encoding NS1 binding factors, according to the manufacturer's instructions. In brief, the CDC25H(α) yeast strain was transformed with the bait plasmid pSos-NS1 and the library DNA purified from the premade libraries, in which human lung cDNAs were conditionally expressed as fusions with a myristoylation membrane localization signal from a GAL1 promoter. A total of 1.09×10^7 ^primary transformants were screened for an interaction, as determined by their ability to grow on minimal synthetic medium in the absence of leucine and uracil in plates containing glucose but not galactose at 23°C. Putative clones were isolated by their ability to grow on minimal synthetic medium in the absence of leucine and uracil in plates containing galactose but not glucose at 37°C. The interaction was verified by retransformation of pSos-NS1 with pMyr cDNA plasmid DNA isolated from the putative clones previously identified.

### Transient transfection and subcellular localization

The NS1 gene from avian influenza virus A/Swine/Colorado/1/77 (H3N2) was kindly provided by ShuZhang Feng. 293FT cells were transfected with pDsRed-GAS8 and pDsRed-GAS8/pEGFP-NS1 using Lipofectamine 2000 (Invitrogen). After twenty-four hours, cells were fixed with 4% paraformaldehyde and washed with PBS. Nuclei were stained with DAPI (Sigma). The subcellular localization of the pDsRed-GAS8 and pEGFP-NS1 fusion proteins was detected using a Zeiss LSM 510 META microscope equipped with a 100× objective lens (Zeiss).

### GST pulldown assays

GST and GST-NS1 proteins were expressed in *Escherichia coli *BL21 induced with isopropyl-β-D-thiogalactopyranoside (IPTG) (Merck). Five milligrams of GST and GST-NS1 were adsorbed from bacterial lysates to MagneGST™ particles, as recommended by the manufacturer (Promega). The immobilized proteins were mixed with 2 mg of 293FT cell extracts containing myc-tagged Gas8 protein in binding buffer (50 mM Tris-HCl, 150 mM NaCl, 1.0%Triton X-100, pH 7.6) containing a protease inhibitor mix (Roche) and incubated for 1 h at room temperature on a rotating platform. Beads were precipitated and washed three times with 500 μl of binding buffer. Proteins bound to GST and GST-NS1 were analyzed by SDS-PAGE, followed by western blot analysis with anti-myc antibody (Invitrogen).

### Immunoprecipitation and immunoblotting

293FT Cells were transfected with pCMV-Tag 2B-Gas8 and pCMV-Myc-NS1 expression plasmids. Following transfection for 48 h, the cells were lysed in RIPA buffer (150 mM NaCl, 1% NP-40, 0.5% deoxycholic acid, 0.1% SDS, 50 mM Tris-HCl, pH 7.5) containing protease inhibitors. Lysates were immunoprecipitated (IP) using anti-FLAG M2 (or anti-Myc) mouse monoclonal antibody (Sigma). Immunoprecipitated proteins were immunoblotted (IB) with anti-myc (or anti-FLAG) antibody. Positive bands were detected with the ECL western blotting detection reagent (Amersham), and the image was visualized with CL-X posure™ film (Pierce). To identify the immunoprecipitated Gas8-interacting proteins, 293FT cells were transfected with pCMV-Tag 2B-Gas8 or pCMV-Tag 2B. Following transfection for 48 h, the cells were lysed in RIPA buffer. Lysates were IPed using anti-FLAG M2 mouse monoclonal antibody, and the immunoprecipitated proteins were resolved by SDS-PAGE and Coomassie stained. The peptide mass fingerprint analysis was performed by the National Center for Biomedical Analysis, Beijing.

### Immunofluorescence and confocal microscopy

CV-1 cells were transfected with pDsRed-GAS8. After 24 hours, the cells were washed with 1 ml of PBS and fixed in methanol:acetone (v/v = 1:1). After PBS washes, rat anti-β-actin serum (Sigma) was applied at a dilution of 1:200, followed by a one-hour incubation at room temperature. The cells were washed with PBS and incubated with fluorescein-conjugated affinipure goat anti-mouse IgG (Zhongshan Goldenbridge Biotechnology Co., China) at a dilution of 1:50. Cells were washed with PBS and examined with a confocal microscope.

## Results

### Isolation of NS1 binding proteins

The CytoTrap system makes use of the yeast *S. cerevisiae *temperature-sensitive mutant strain cdc25H. The strain grows normally at 23°C, but does not grow at 37°C. The CytoTrap system is based on the ability of the human Sos protein, which is the human homologue of the yeast CDC25 gene, to complement the cdc25 defect and to activate the yeast Ras signaling pathway. Expression of hSos and its subsequent localization to the plasma membrane allows the cdc25H yeast strain to grow at 37°C. The localization of Sos to the plasma membrane occurs through the interaction of two-hybrid proteins. We used a pSos-NS1 fusion protein to screen a human lung cDNA plasmid library, in which cDNA-encoded proteins were conditionally expressed as translational fusions with a pMyr. Expression of the pMyr fusion proteins is induced in the presence of galactose and repressed by glucose. Twenty-four library plasmids were isolated from 1.09 × 10^7 ^transformants based on their ability to grow on minimal synthetic medium in the absence of leucine and uracil in plates containing galactose but not glucose at 37°C. Further identification of putative positive plasmids was performed by co-transformation with pSos-NS1, and 15 out of 24 were confirmed as positive plasmids. After DNA sequencing, 8 clones of different gene were obtained. Here, we report the analysis of one of the human cDNAs isolated from these library plasmids that encodes growth arrest-specific gene 8.

### NS1 interacts with Gas8 in vitro and in vivo

**Figure 1:**
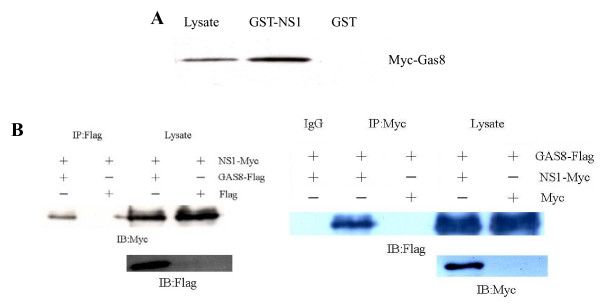
***In vitro *and *in vivo *interaction between NS1 and Gas8**. **A **GST pull-down analysis. Lysates from cells expressing myc-Gas8 were mixed with GST or GST-NS1, and bound proteins were analyzed by anti-myc western blot. Left lane, cell lysate; middle and right lanes, proteins bound to GST-NS1 and GST, respectively. Gas8 was precipitated by a GST-NS1 fusion protein but not by GST. **(B) **Western blot of NS1 and Gas8 co-IPs. Left: Cells were co-transfected with a plasmid expressing myc-tagged NS1 and either a plasmid expressing FLAG-tagged Gas8 or the empty vector. Cell lysates were IPed with anti-FLAG antibody (left two lanes) or run on a gel without immunoprecipitation (right two lanes). Samples were probed with anti-myc and anti-FLAG antibody. Right: IP with anti-myc antibody and IB with anti-FLAG and anti-myc antibody. The results showed that NS1 could be precipitated with Gas8 and Gas8 could also be precipitated with NS1.

To confirm the interaction between NS1 and Gas8 suggested by the two-hybrid results, two experiments were performed. First, GST pull-down assays we performed to find proteins that bind to a GST-NS1 protein in 293FT cell lysates expressing myc-tagged Gas8. Gas8 was efficiently precipitated by a GST-NS1 fusion protein but not by GST (Fig. 1A), indicating that NS1 interacts with Gas8 *in vitro*. Next, the interaction of NS1 and Gas8 *in vivo *was investigated by co-immunoprecipitation (co-IP). When lysates of 293FT cells expressing FLAG-tagged Gas8 were immunoprecipitated with anti-FLAG antibodies, myc-tagged NS1 protein was detected in the precipitate. Similarly, Gas8 protein could also be precipitated by NS1 (Fig. 1B).

### Protein-protein interaction domains

**Figure 2:**
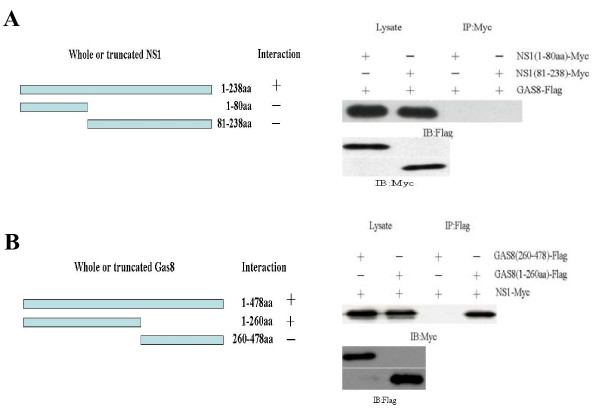
**Protein-protein interaction domains**. **(A) **Mapping of the NS1 domain interacting with Gas8. 293FT cells were transfected with pCMV-Tag 2B-Gas8 and one of its truncated derivatives: pCMV-myc-NS1_1-80_, pCMV-myc-NS1_81-238_, or pCMV-myc. After incubation for 36 h, cell lysates were IPed with anti-myc antibody and probed with anti-FLAG and anti-myc antibody. **(B) **Schematic representation of Gas8 proteins. Polypeptides corresponding to Gas8_1-478_, Gas8_1-260_, and Gas8_260-478 _are shown. The corresponding amino acids are indicated on the right. 293FT cells were transfected with pCMV-myc-NS1 and one of its truncated derivatives: pCMV-Tag 2B-Gas8_1-260_, pCMV-Tag 2B-Gas8_260-478_, or pCMV-Tag 2B. Cell lysates were IPed with anti-myc antibody and probed with anti-FLAG and anti-myc antibody. +: protein-protein interaction; -: no protein-protein interaction.

To identify the region of NS1 protein involved in the interaction with Gas8 protein, *in vivo *binding experiments were carried out. 293FT cells were transfected with the plasmids pCMV-Tag 2B-Gas8 and pCMV-myc-NS1_1-238 _or one of its truncated derivatives: pCMV-myc-NS1_1-80_, pCMV-myc-NS1_81-238_, or pCMV-myc. When lysates of 293FT cells transfected with the different plasmids mentioned above were immunoprecipitated with anti-myc antibodies, no FLAG-tagged Gas8 protein was detected in the cells expressing truncated NS1 (Fig. 2A). The protein-protein interaction domain of Gas8 was identified by IP assays, as described above, this time using Gas8 deletion mutants. Gas8_1-260 _interacted with NS1. In contrast, no interaction with Gas8_260-478 _was observed (Fig. 2B).

### Gas8 and NS1 proteins co-localize

**Figure 3:**
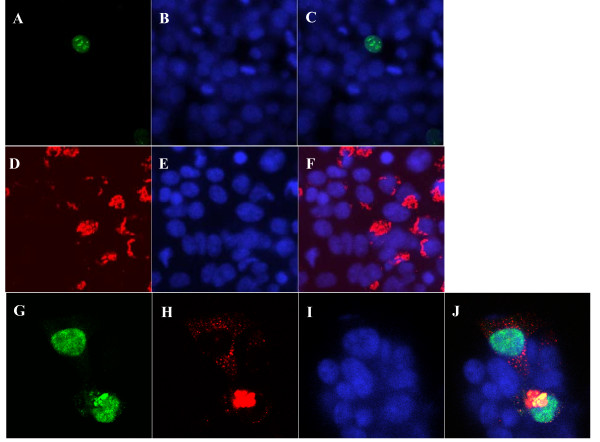
**Confocal micrographs of NS1 and Gas8 in 293FT cells**. Panels **(A) **and **(G)**: GFP. Panels **(D) **and **(H)**: DsRed. Panels **(B)**, **(E)**, **(I)**: DAPI. Panels **(C)**, **(F)**, **(J)**: merged images. Top row: cells transfected with pEGFP-NS1 showing nuclear localization. Middle row: cells transfected with pDsRed-GAS8 showing juxtanuclear localization. Bottom row: co-expression of NS1 and Gas8 showing juxtanuclear localization.

Based on the two-hybrid and *in vitro *binding results, we hypothesized that Gas8 and NS1 interact *in vivo*. If this interaction is physiologically relevant, we would expect the two proteins to occupy the same intracellular compartment. To test this prediction, a Gas8-DsRed and an NS1-GFP fusion were co-expressed in 293FT cells. The Gas8 protein has been reported to localize to the Golgi apparatus [21]. The NS1 protein has been found in large foci in the nucleus [23,24], a localization that was confirmed in 293FT cells (Fig. 3). When both proteins were co-expressed, fluorescently tagged Gas8 and NS1 exhibited juxtanuclear co-localization. These results suggest that Gas8 can modify the location of NS1.

### Gas8 interacts with actin, myosin, and drebrin

**Figure 4:**
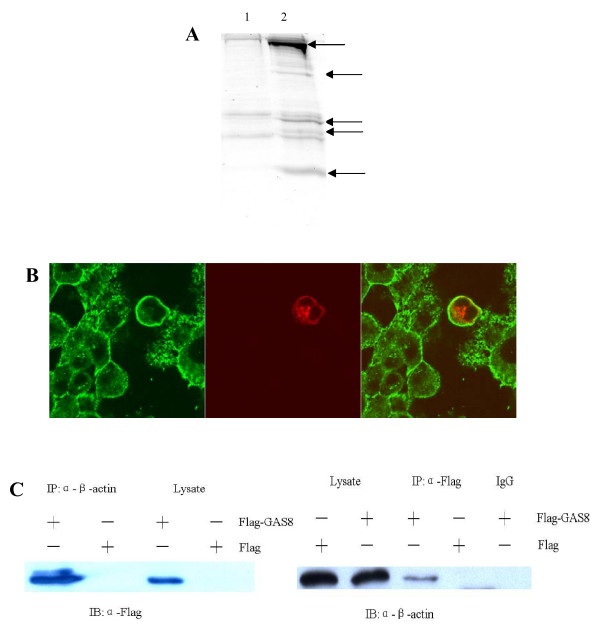
**Interaction between Gas8 and β-actin**. (A)SDS-PAGE analysis of proteins that co-IPed with Gas8. 293FT cells were transfected with pCMV-Tag 2B-GAS8 or pCMV-Tag 2B, and cell extracts were immunoprecipitated with anti-FLAG antibody. The gels were Coomassie stained. 1: pCMV-Tag 2B/293FT, 2: pCMV-Tag 2B-GAS8/293FT. Arrows indicate proteins that co-IPed with Gas8. **(B) **Gas8 co-localizes with β-actin at the plasma membrane. CV-1 cells were transfected with pDsRed-GAS8. After 24 h, the cells were fixed and probed with an antibody against β-actin. The merged image shows co-localization of Gas8 (red) and β-actin (green) at the plasma membrane. **(C) **Interaction of Gas8 and β-actin proteins *in vitro*. 293FT cells were transfected with pCMV-Tag 2B-GAS8 (FLAG-Gas8) or pCMV-Tag 2B (FLAG). Soluble cell lysates were IPed with anti-FLAG or anti-β-actin antibody and probed with anti-FLAG (left) or anti-β-actin antibody (right).

To find cellular proteins that interact with Gas8, co-immunoprecipitated proteins were identified by peptide mass spectrometry (Fig. 4A). Peptide fingerprinting identified the proteins myosin-9, drebrin E2, and actin. The actin-binding protein drebin has been localized to the apical plasma membrane together with a pool of submembranous F-actin, which is hypothesized to modulate actin-myosin interactions in dendritic spines [25,26]. Myosin 9 may function in intracellular vesicle transport [27]. CV-1 cells transfected with pDsRed-GAS8 were analyzed by confocal microscopy and immunofluorescence using anti-β-actin antibody. The results showed that Gas8 and β-actin co-localize at the plasma membrane, but not in the cytoplasm (Fig. 4B). Their association was confirmed by co-IP (Fig. 4C).

## Discussion

Gas8 was obtained from a two-hybrid screen for cytoplasmic interactions with the influenza A protein NS1. The interaction was confirmed *in vitro *and *in vivo *with GST-pulldown and co-IP assays. Identification of the NS1 domain that interacts with Gas8 revealed that neither the RNA binding domain nor the effector domain of NS1 alone could interact with Gas8, in contrast to a previously reported NS1-interacting protein[28]. Deletion analysis revealed that the N-terminal 260 amino acids of Gas8 were able to interact with NS1. This domain corresponds to IMAD and GMAD domain.

Examination of the localization of Gas8 protein in cells revealed that two types of localization exist: Golgi and cytoplasmic. This phenomenon was even observed in the same types of cells. The Golgi apparatus localization is dependent on intact microtubules and is cell-cycle regulated, as Gas8 is dispersed throughout the cytoplasm as cells progress through mitosis[21].

Since the function of Gas8 has not yet been determined, the effect of the association between Gas8 and NS1 on virus infection is unknown. The mammalian Gas8 gene is a possible tumor suppressor that was previously identified as one of several genes that are up-regulated upon growth arrest[19]. Cell proliferation was not inhibited when CV-1 and DU145 cells were transfected with the recombinant plasmid pCMV-Tag 2B-GAS8. Gas8 was expressed in HeLa, CV-1, A549, and DU145 cells. NS1 inhibited the maturation of GAS8 mRNA. This is consistent with the fact that transient expression of NS1 in mammalian cells leads to retention of poly(A) RNA in the nucleus and inhibition of pre-mRNA splicing[1,29]. In order to study the function of Gas8, we attempted to abrogate the expression of Gas8. Unfortunately, an effective target for RNAi was not found.

We identified proteins that interact with Gas8 by immunoprecipitation and found that Gas8 co-localizes with actin at the plasma membrane, but not in the cytoplasm. We speculate that this may be the result of its association with drebrin E2, which is the only protein of the myosin-9-drebrin E2-actin complex reported to be localized to the apical plasma membrane with actin[26]. However, successful expression of the drebrin E2 protein was not achieved, and therefore a direct interaction between Gas8 and drebrin still needs to be confirmed. Drebrin has been reported to modulate actin-myosin interactions in dendritic spines, and myosin 9 may function in intracellular vesicle transport[26,27]. Therefore we examined the effect of overexpression of Gas8 on the maturation of progeny virions and found that Gas8 could arrest the production of H3N2 progeny virions from cells.

Gas8 has been reported to interact with Rab3B. Rab3 subfamily proteins are enriched in neuronal and secretory cells, where they control regulated exocytosis through the interaction with the Rab3 effector proteins Rabphilin-3, Rim1/2, and Noc2 [30]. In fibroblasts and epithelial cells, Rab3B is involved in the transport of low-density lipoprotein receptors, vesicular stomatitis virus glycoprotein, and polymeric immunoglobulin receptors [31-33]. Overexpression of Noc2 inhibited the cell-surface transport of basolateral vesicular stomatitis virus glycoprotein. Consistent with the functions of these Gas8-associated proteins and the binding detected between Gas8 and drebrin, actin, and myosin, we speculate that Gas8 may affect the release of H3N2 progeny virions.

## Competing interests

The authors declare that they have no competing interests.

## Authors' contributions

LXZ and LX performed analysis of interaction between Gas8 and NS1 and wrote the manuscript. XWZ, QYZ, XDZ and PTH conceived the studies and participated in experimental design and coordination. CFZ isolated Gas8 from the library. ZXY and XHZ carried out viral replication in cell culture. MBY and YYZ participated in abrogation the expression of Gas8. All authors read and approved the final manuscript.
